# Salivary Protein and Electrolyte Profiles during Primary Teeth Eruption: A Cross-Sectional Study

**DOI:** 10.3390/diagnostics13071335

**Published:** 2023-04-03

**Authors:** Mina Ahmadian, Sara Maleki Kambakhsh, Nahid Einollahi, Saber Babazadeh, Maryam Tofangchiha, Giuseppe D’Amato, Romeo Patini

**Affiliations:** 1 School of Dentistry, Qazvin University of Medical Sciences, Qazvin 34199-15315, Iran; 2 Dental Caries Prevention Research Center, Qazvin University of Medical Sciences, Qazvin 34199-15315, Iran; 3 Department of Clinical Laboratory Sciences, Faculty of Allied Medical Sciences, Tehran University of Medical Sciences, Tehran 14197-33141, Iran; 4 Department of Community Oral Health, School of Dentistry, Mashhad University of Medical Sciences, Mashhad 91779-48959, Iran; 5 Unicamillus International University of Health and Medical Sciences, 00131 Rome, Italy; 6 Department of Head, Neck and Sense Organs, School of Dentistry, Catholic University of Sacred Heart, 00135 Rome, Italy

**Keywords:** tooth eruption, saliva, proteins, tooth, deciduous, sodium, potassium

## Abstract

This study aimed to assess the qualitative changes in the saliva during the process of primary teeth eruption. This cross-sectional study was conducted on 147 children from 2 to 48 months, of which 49 were in group A (no erupted primary teeth), 53 were in group B (at least one active erupting primary tooth), and 45 were in group C (eruption of all 20 primary teeth was completed). Salivary proteins were evaluated by sodium dodecyl sulfate electrophoresis with polyacrylamide gel, while the concentrations of salivary sodium, potassium, chloride, and calcium ions were evaluated by ion selective electrodes. The data were analyzed using ANOVA and Bonferroni tests (alpha = 0.05). The concentration of proteins with molecular weights of 20–30 KDa was significantly higher in group A, and it gradually decreased with age. The concentration of proteins with molecular weights of 50–60 KDa in group B was significantly lower than those of groups A and C. The calcium ion concentration in group A was significantly higher than that of the other groups. The concentration of potassium ions was minimal in group C. The proteins and electrolyte profiles of the subjects’ saliva changed in the process of primary tooth eruption. The highest concentrations of proteins such as statherin, histatin, P-B peptide, and cystatin and the lowest concentrations of proteins such as amylase were present in group B.

## 1. Introduction

Tooth eruption is a physiological process during which the teeth erupt into their position in the dental arch. This is a highly regulated process controlled by gene interactions. Bone loss occurs by the activity of osteoclasts, and the presence of dental follicles is the biological basis of tooth eruption [1]. The formation and eruption of primary teeth occur following consecutive mutual cell/tissue interactions during fetal development and also after birth [2]. 

Tooth eruption is a natural process which is often associated with no or minimal complications. Nonetheless, some infants show signs of systemic complications such as a mild fever (usually lower than 101 °F), diarrhea, dehydration, hyper-salivation, skin rashes, and gastrointestinal problems [3]. Some children put their fingers into their mouths, and some become irritable and bad-tempered during this period. Gingival inflammation before the completion of tooth eruption may cause pain, which often subsides within a couple of days. Further, based on a past study completed by Iavarone, it appears that in complex process of tooth eruption, the peptide/protein profile of gingival cervicular fluid changes dramatically [4]. 

Conditions such as the presence of an eruption or dentigerous cystsand anomalies related to “tooth” size or number (supernumerary teeth), or facio skeletal anomalies such as clefts can alter this physiologic pattern [5]. 

In the first year after birth, significant changes occur in an infant’s oral environment. Some of these changes may be related to the eruption of primary teeth. 

Biochemically, salivary proteins can provide valuable biological information and serve as potential biomarkers for the detection of oral and systemic conditions. Thus, knowledge about the salivary proteins related to each cycle of life and the influential factors in this regard is imperative [6,7]. 

Electrolytes, especially sodium and potassium, present in biological fluids directly affect protein balances. Evidence shows that diseases, dental interventions, and biological conditions can change the ionic balance of the saliva [8]. 

Considering the non-invasive nature of saliva collection, in the case of being familiar with the salivary profiles of different stages, it can serve as a valuable method for the early detection of diseases [6,7]. To better understand the effects of tooth eruption on the quality of saliva, this study aimed to assess the qualitative changes in the salivary proteins and ions in the process of primary tooth eruption in 2- to 48-month-old children.

## 2. Materials and Methods

This cross-sectional study was conducted on the salivary samples of 2- to 48-month-old children. The study was approved by the ethics committee of the Qazvin University of Medical Sciences (IR.QUMS.REC.1396.272).

The total sample size was calculated at 135 (for each group, at least 45) for 3 equal groups, and the SD (0.27) concentrations of the major salivary proteins were assumed from a previous study [9] considering an alpha of 5% and a study power of 80% using PASS 11.

The children were selected among those presenting at health centers or attending the kindergartens in Qazvin city. The parents were informed about the study and willingly signed informed consent forms before letting their children participate in the study. The parents were requested to fill out a questionnaire regarding the demographic information, oral hygiene measures, medical history, medication intake, and growth and development of their children. Clinical examinations were performed by a pediatric dentist using a dental mirror and a spotlight without radiography. The examiner marked the erupted and erupting teeth on the questionnaire. For children with erupting teeth, the associated signs and symptoms were also recorded. 

The inclusion criteria were healthy infants and children with no medical problems (anomalies or syndromes) (based on the questionnaire), aged between 2 and 48 months, with normal growth patterns, and whose parents consented to their participation in the study [10].

The exclusion criteria were a history of acute and chronic systemic diseases (in the past one month), a history of medication intake in the past 2 weeks, the presence of dental caries (if, during the clinical examination, the dentist expressed doubt about presence of caries or the need to take dental radiographs, the child was excluded), the presence of periodontal disease or gingival inflammation, and the consumption of food or water within one hour prior to saliva sampling.

To assess protein/ion changes before, during, and after the active tooth eruption process, the children were divided in three groups:

Group A: infants with no primary tooth

Group B: children with 1 to 19 primary teeth who had at least one actively erupting tooth (active eruption was defined as visibility of the cusp tip or incisal edge of the respective tooth in the oral cavity)

Group C: children who had all 20 primary teeth in their oral cavity

All saliva samples were collected within a 2 h period between 10 a.m. and 12 p.m. to minimize inter-individual differences due to circadian rhythms. To ensure the collection of unstimulated saliva samples, the participants were asked to refrain from eating and drinking for one hour prior to saliva collection. The unstimulated saliva samples were collected by a 1 mL needleless syringe according to previous studies [9,10,11,12,13]. After sampling, the parents received oral hygiene and nutritional instructions regarding their children.

The collected saliva was transferred into capped microtubes and refrigerated to prevent hydrolysis of the salivary proteins. After the sample size was reached, the samples were transferred to the laboratory of the Immunology Department of the School of Medicine, Qazvin University of Medical Sciences. All microtubes were allocated a code which matched the coding of the questionnaires. The samples were then frozen at −20 °C and kept at −80 °C until the appropriate analyses were performed. For the analyses, the samples were thawed at room temperature and then centrifuged (Eppendorf centrifuge 5415, Germany) at 3000 rpm for 10 min. By doing so, all particles possibly present in the saliva, such as bacteria, were precipitated to the bottom of the microtubes. The supernatant was gently and carefully collected by a sampler and transferred into another microtube.

SDS-PAGE electrophoresis (Bio-Rad, Richmond, VA, USA) and Coomassie blue staining were used to analyze the concentrations of the salivary proteins according to previous studies [14,15]. The densitometric scans of the SDS-PAGE gels were performed using a scanner (CanoScan LiDE 210, Canon, CO, USA), and the intensities of the protein bands were analyzed by Image J software. The ion-selective electrodes were used to assess the salivary ions.

The data were analyzed by ANOVA, followed by the Bonferroni post-hoc tests (for pairwise comparisons) using SPSS version 24 at a 0.05 level of significance.

## 3. Results

A total of 147 children aged between 2 and 48 months participated in this study. Of them, 49 were allocated to group A (no eruption of primary teeth), 53 to group B (at least one erupting primary tooth), and 45 to group C (having all 20 primary teeth). Table 1 presents the demographic information of children in the three groups.

Group A: no eruption of any primary teeth; group B: having at least one erupting tooth; and group C: having all 20 primary teeth.

Table 2 presents the signs and symptoms of the children with at least one erupting tooth in group B. As shown, the most commonly reported symptoms were putting fingers and hands into the mouth and hyper-salivation.

After electrophoresis and Coomassie blue staining, nine bands, according to the molecular weights of the proteins, were detectable on the gel. Table 3 indicates the mean concentrations of the proteins identified in the three groups.

The ANOVA tests revealed significant differences in the concentrations of proteins with molecular weights of <10, those between 15 and 20, those between 20 and 30, and those between 50 and 60 KDa among the three groups (*p* < 0.05). Thus, pairwise comparisons were performed using the Bonferroni post hoc test (Table 4).

**Figure 1:**
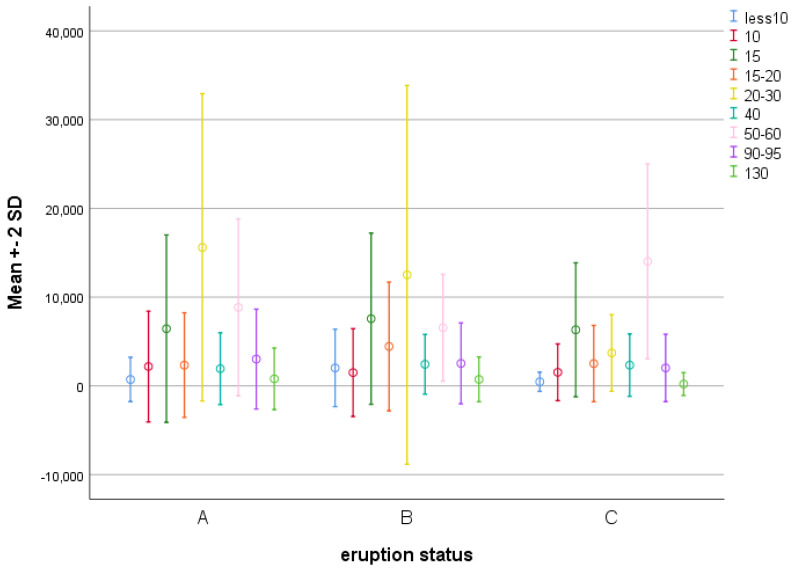
Means ± SD concentrations of proteins with different molecular weights in the three groups.

The concentrations of proteins with molecular weights of < 10 KDa in group B were significantly higher than those of groups A and C (*p* < 0.001). In addition, the concentrations of proteins with molecular weights of between 15 and 20 KDa in group B were significantly higher than those of groups A and C (*p* = 0.002 and *p* = 0.008, respectively). The concentrations of proteins with molecular weights of between 20 and 30 KDa in group C were significantly higher than those of groups A and B (*p* < 0.001). The concentrations of proteins with molecular weights of between 50 and 60 KDa in group B were significantly lower than those of groups A and C (*p* = 0.02 and *p* < 0.001, respectively). The values in group C were significantly higher than those in group A (*p* < 0.001) (Figure 1).

Table 5 presents the mean concentrations of sodium, potassium, chloride, and calcium ions in the three groups. As shown, the three groups were significantly different regarding the concentrations of potassium and calcium ions (*p* < 0.001). Thus, pairwise comparisons using the Bonferroni test were performed.

The results showed that the concentrations of potassium in group C were significantly lower than those of groups A (*p* < 0.001) and B (*p* < 0.001). However, the concentrations of potassium ions were not significantly different in groups A and B (*p* = 0.797).

**Figure 2:**
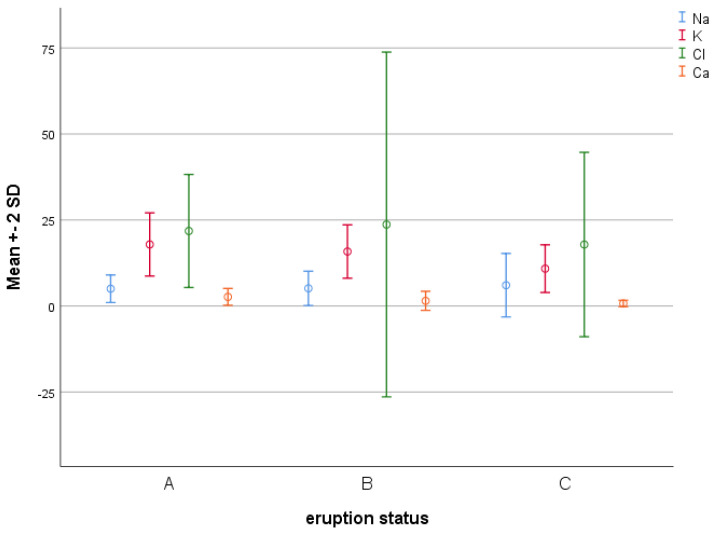
Means ± SD concentrations of sodium, potassium, chloride, and calcium ions in the three groups.

The concentrations of calcium ions in group A were significantly higher than those of groups B (*p* < 0.001) and C (*p* < 0.001). Further, the concentrations of calcium ions in group C were significantly lower than those in group B (*p* = 0.002) (Figure 2).

## 4. Discussion

Saliva is composed of a wide range of proteins, peptides, electrolytes, and nucleic acids. Considering the non-invasive collection of saliva compared with blood samples, it can be used to assess the important biochemical reactions that occur in the oral environment and may serve as a valuable source for the detection of different disease conditions [16]. This study assessed the concentrations of salivary proteins and ions in the process of the eruption of primary teeth in 2- to 48-month-old children, and any condition that could interact with the normal eruption process was excluded. The results indicated a change in the proteins and electrolyte profiles of the saliva during the process of the eruption of primary teeth such that significant differences were noted among the three groups for the concentrations of proteins with different molecular weights, as well as calcium and potassium ions. The maximum levels of proteins with molecular weights of <10 KDa and those of between 15 and 20 KDa were noted in the saliva samples from children with erupting primary teeth. However, children with no erupted teeth and those with complete primary dentition had no significant difference in this respect.

Different proteins with molecular weights of <10 KDa have been identified in saliva, such as statherin (4–6 KDa), histatin (4–6 KDa), and P-B peptide (6 KDa) [17]. The results of some previous studies were in line with the present findings, and there have been reported changes in the levels of histatin with age. Manconi et al. [10] reported a significant increase in salivary levels of histatin in 7–12-month-old infants compared with 0–6-month-old infants. Messana et al. [9] evaluated the salivary protein profiles of infants and adults, and they found that histatin appeared at 7 months of age for the first time. They attributed the changes in the concentrations of histatin to its wound-healing properties and tissue formation. Oudhoff et al. [18] highlighted the important biological role of salivary histatin in wound-healing. Considering the onset of tooth eruption at 7 months of age and the confirmed role of histatin in wound-healing, the increased concentrations of histatin may be attributed to its possible role in gingival healing following tooth eruption [19].

Statherin is another salivary protein with a molecular weight of <10 KDa. It has a pivotal role in the mineral dynamics on a tooth’s surface. Messana et al. [9] reported that statherin appears in the saliva of infants at 7 months of age. However, Manconi et al. [10] reported that the expression of statherin was not affected by age. P-B peptide is another salivary protein with a molecular weight of < 10 KDa (6 KDa). The results of Messana et al. [9], Manconi et al. [10], and Morzel et al. [20] regarding this protein were in agreement with the present findings. They indicated increases in the salivary levels of this peptide with age. This peptide has been reported to be a regulator of the immune system. It suppresses the host’s inflammatory response by regulating the release of cytokines, and it exerts antioxidant effects by detoxifying oxygen free radicals. It appears that the role of saliva in inflammatory processes changes with the eruption of teeth into the oral cavity and with alterations in the microbial flora [9,10,20].

As mentioned earlier, the maximum concentrations of proteins with weights of 15–20 KDa were noted in the children with erupting primary teeth. The cystatin family of proteins (S, SN, and SA) is highly important in this molecular weight range. Different cystatin proteins have molecular weight sin the range of 11 to 17 KDa [21].

SN and SA salivary cystatins inhibit lysosomal cathepsins, control proteolytic reactions, and inhibit the activity of cysteine proteases. Thus, they are believed to play an important role in the degradation of periodontium. However, type S cystatin has a completely different role. It can form greater and more extensive bonds with calcium and apatite crystals, and thus, it is believed to have a primary role in the regulation of the mineral content of teeth. Dickinson et al. [22] reported increases in the levels of cystatin in 2- to 9-month-old infants. Other studies have also reported the emergence of this protein in the saliva in children at approximately one year of age [9,10,23].

The molecular weights of acidic and alkaline proline-rich proteins (PRPs) range from 10–40 KDa; some of them are in the weight range of 15–20 KDa. Inzitari et al. [11] showed that acidic PRPs were present in the saliva samples of preterm infants. These proteins are among the structural proteins of the oral cavity, and they play important roles in the early days of life. In addition, evidence shows that acidic PRPs play a role in the regulation of salivary calcium ions and can bond to hydroxyapatite. Thus, they participate in the formation of pellicle on tooth surfaces. Further, it has been suggested that they are important regulators of the colonization of salivary bacteria [11,24,25]. In the study by Inzitari et al. [11], the concentrations of acidic PRPs in infants were lower than those of adults, and their concentrations increased with age. These results were in agreement with those of Manconi et al. [10]. The concentrations of these proteins in 0–6-month-old infants were significantly lower than those of 25–36 and 37–48-month-old children, and they increased with age [11,12].

In the present study, the maximum concentrations of proteins with molecular weights of 20–30 KDa were noted in the infants without any eruption of primary teeth, and these concentrations gradually decreased with age such that their concentrations in group A had a significant difference compared to those in group C (children with complete primary dentition). Morzel et al. [20] discussed that tooth eruption had a moderate effect on the frequency of some peptides such as PRPs. In the present study, the lowest concentrations of proteins with molecular weights of 50–60 KDa were noted in the infants with erupting primary teeth, and the difference in this respect was significant compared to the other groups.

Amylase is a protein with a molecular weight ranging from 55–60 KDa. Morzel et al. [20], Böttcher et al. [26], and Wan et al. [27] reported increases in the levels of amylase in 3–6- and 18–24-month-old babies. However, Gleeson and Cripps [28] reported an insignificant change in the concentration of amylase during childhood [15,26,27,28]. In a study by Tabbara [15], the levels of amylase were minimal in edentulous adults, and they were at maximum levels in children with complete primary dentition. This finding indicates that there are higher concentrations of amylase in individuals with complete dentition, and it highlights the role of amylase in the prevention of bacterial adhesion to tooth surfaces. Bellavia et al. [29] evaluated the activity of amylase in neonates (<1 month of age) and older infants and concluded that it had lower activity in neonates, and its activity gradually increased during the first year of life. In the present study, increases in the concentrations of proteins with weights of 50–60 KDa were noted during the process of the eruption of primary teeth, which continued until complete primary dentition was achieved. However, it should be noted that alpha amylase is one of the important proteins in this molecular weight range, and other proteins in this range should also be investigated in further studies.

Ruhl et al. [30] reported that the overall profile of salivary proteins and glycoproteins is relatively stable during the childhood period, with some exceptions. For instance, mucin and albumin showed some alterations. The albumin concentration had a shift approximately 1 month prior to the eruption of the first primary tooth. Since albumin is derived from serum, it enters the oral cavity through the permeable mucosa or the gingival sulcus; this may explain the low concentrations of albumin in infants. Tabbara [15] also reported changes in the concentration of albumin. Albumin has a 67 KDa molecular weight. It is secreted into the gingival crevicular fluid, and its concentration increases by an increase in the number of erupted teeth. Tabbara [15] indicated maximum concentrations of albumin in children with maximum numbers of teeth, while its minimum concentrations were noted in children and adults with no teeth.

Mucin, with a 200–300 KDa molecular weight, is another important salivary protein that is present in two forms of soluble secretory proteins (MUC5 and MUC7) and another form related to the epithelial membrane (MUC1). This protein plays a role in the formation of pellicle, a protective layer over a tooth’s enamel, and it serves as a lubricant in the mucosa and preserves moisture. sIgA is another protein with a 380 KDa molecular weight, though it was not evaluated in this study due to its high molecular weight, and it requires more advanced analyses.

Changes in the peptide profile can occur for several reasons. Many alterations occur in the first couple of months after birth that affect the oral environment, such as the eruption of primary teeth and changes in the diet during this period. For example, tooth eruption through the gingiva results in the release of gingival fluid into the saliva [31].

The present results indicated significant differences in the concentrations of potassium and calcium ions among the three groups. Calcium ions had the highest concentrations in the saliva samples of infants with no primary teeth, and they gradually decreased until the completion of primary dentition was achieved. Potassium ions had the lowest concentrations in the saliva samples of children with complete primary dentition, and there were significant differences compared to the other two groups. The differences in sodium and chloride ion levels were not significant among the three groups.

It has been claimed that the ionic and, particularly, calcium contents in saliva are influenced by the protein content. Acidic PRPs and statherin are among the proteins responsible for the regulation of calcium ions. Since tooth mineralization is a dynamic process and mineralization of the teeth continues following their eruption, it appears that the calcium reduction in the complete dentition group may have been due to its deposition on tooth surfaces and its participation in the mineral structure of teeth. Sympathetic and parasympathetic stimuli can also change the electrolyte composition of the saliva [32,33]. De Oliveira et al. [32] showed that increases in saliva volume with age (>15 years of age) can be associated with changes in sodium, potassium, and chloride ion levels. Deshpande et al. [7] evaluated 3–16-year-old children and reported linear increases in calcium concentrations from the primary dentition to permanent dentition periods, which was not significant. The concentration of potassium was at its maximum level during the mixed dentition period.

Studies on salivary electrolyte changes during physiological processes are limited, and the available ones have mainly focused on the effects of systemic diseases and pathological conditions in this respect. For instance, Singh et al. [34] compared the salivary electrolytes of children with Down syndrome in comparison with normal controls. They reported higher levels of sodium, potassium, chloride, and calcium ions in the Down syndrome children compared to the controls. They suggested the possible reason to be the increased activity of salivary carbonic anhydrase. Rodrigues et al. [35] evaluated healthy controls and those undergoing dialysis. They reported greater changes in the levels of calcium in the dialysis patients. The potassium levels were not significantly different between the two groups. They concluded that the secretion of albumin into the saliva was positively correlated with increases in calcium and potassium electrolytes.

Shirzaii and Heidari [36] evaluated the chemical composition of the saliva in diabetic patients, and they reported increases in potassium levels and reductions in sodium levels compared with healthy controls. The differences in salivary calcium levels were not significant. Bang et al. [37] found a significant and direct correlation between the potassium concentrations in gingival crevicular fluid and the mean pocket depth. Considering the increases in pocket depth in children with erupting primary teeth compared with those with complete dentition, the differences in the potassium concentrations in this study can be justified.

This present study has several limitations. Saliva collection in infants is more challenging than in older children and requires a parent’s cooperation, which can be considered in a future study’s design. In the present study, salivary proteins were evaluated by electrophoresis because of limited facilities and financial resources. Future studies are recommended to use mass spectrometry, chromatography, and specific kits for more accurate protein identification.

The results obtained from this study show that changes in the protein and electrolyte levels during the dynamic process of tooth eruption are consistent with nutritional and gestational needs. Further, the proteins related to the inflammatory process of bone remodeling during active tooth eruption and the proteins/ions that regulate tooth mineralization and maturation changed dynamically.

## 5. Conclusions

The protein and electrolyte profiles of the saliva change during the process of primary tooth eruption.

The highest concentrations of proteins such as statherin, histatin, P.B peptide, and cystatin and the lowest concentrations of amylase were present in the active tooth eruption group. PRPs were evaluated and showed high concentrations in children with complete primary tooth eruption. Potassium and calcium concentrations decreased with increasing age.

## Figures and Tables

**Table 1:** Demographic information of the children in the three groups.

Variable		Age (Month)	Weight (kg)	Gender		Total (%)
	Group	Mean ± Standard Deviation	Mean ± Standard Deviation	Male Number (%)	Female Number (%)	
A		5.17 ± 6.16	6.59 ± 8.59	25 (51)	24 (49)	49 (100)
B		7.16 ± 14.07	1.95 ± 10.26	32 (60.4)	21 (39.6)	53 (100)
C		8.33 ± 42.11	2.19 ± 14.17	18 (40)	27 (60)	45 (100)

**Table 2:** Signs and symptoms of children in group B with at least one erupting tooth.

Clinical Signs and Symptoms	Number (%)
Gastrointestinal complications	24 (46.15)
Gingival swelling or redness	27 (51.92)
Perioral redness	8 (15.38)
Irritability	42 (80.76)
Hyper-salivation	45 (86.53)
Putting hand in the mouth	50 (96.15)
Skin rashes	4 (7.69)
Malaise	16 (30.76)
Fever	35 (67.30)

**Table 3:** Mean concentrations of the proteins in the three groups.

Molecular Weight (KD)	A	B	C	*p*-Value *
	Mean ± Standard Deviation	Mean ± Standard Deviation	Mean ± Standard Deviation	
**<10**	1228.80 ± 698.15	2181.35 ± 2021.60	548.79 ± 463.13	**<0.001**
**10**	3082.02 ± 2201.19	2473.94 ± 1489.30	1541.47 ± 1455.47	0.261
**15**	5765.84 ± 6933.28	4821.13 ± 7565.93	3607.24 ± 6122.51	0.375
**20–15**	2910.68 ± 2313.71	3629.41 ± 4440.93	2174.24 ± 2486.53	**<0.001**
**30–20**	9399.27 ± 15,960.54	10,672.66 ± 12,512.34	2143.05 ± 3643.56	**<0.001**
**40**	1985.90 ± 1944.01	1684.75 ± 2431.67	1780.50 ± 2326.70	0.378
**60–50**	5010.35 ± 9048.58	3010.74 ± 6554.44	5547.18 ± 13,961.51	**<0.001**
**95–90**	2776.19 ± 2962.21	2278.19 ± 2531.97	1921.05 ± 2001.25	0.169
**>130**	1723.18 ± 774.50	1260.46 ± 725.84	651.98 ± 211.23	0.094

* ANOVA; Group A: no eruption of any primary teeth; group B: having at least one erupting tooth; and group C: having all 20 primary teeth.

**Table 4:** Pairwise comparisons of the three groups regarding the concentrations of proteins with different molecular weights.

Molecular Weight	Group	Mean	*p*-Value *
**<10**	A vs. B	−1323.44 ± 306.42	**<0.001**
	A vs. C	235.02 ± 327.96	1
	B vs. C	1558.46 ± 323.68	**<0.001**
**20–15**	A vs. B	−2127.21 ± 602.27	**0.002**
	A vs. C	−172.81 ± 644.61	1
	B vs. C	1954.39 ± 436.20	**0.008**
**30–20**	A vs. B	3448.20 ± 1714.10	0.139
	A vs. C	12,316.98 ± 1834.60	**<0.001**
	B vs. C	8868.78 ± 1810.66	**<0.001**
**50–60**	A vs. B	2494.14 ± 907.53	**0.020**
	A vs. C	−4912.93 ± 971.33	**<0.001**
	B vs. C	−7407.07 ± 958.65	**<0.001**

* Bonferroni post hoc test; Group A: no eruption of any primary teeth; group B: having at least one erupting tooth; and group C: having all 20 primary teeth erupted.

**Table 5:** Mean concentration of sodium, potassium, chloride, and calcium ions in the three groups.

Electrolyte	A	B	C	*p*-Value *
	Mean ± Standard Deviation	Mean ± Standard Deviation	Mean ± Standard Deviation	
Sodium	2.15 ± 4.81	2.53 ± 5.14	4.58 ± 6.16	0.114
Potassium	4.13 ± 16.90	3.88 ± 16.01	3.79 ± 11.10	**<0.001**
Chloride	8.06 ± 21.21	24.55 ± 23.60	13.25 ± 17.45	0.22
Calcium	1.22 ± 2.64	1.38 ± 1.49	0.46 ± 0.71	**<0.001**

* ANOVA; Group A: no eruption of any primary teeth; group B: having at least one erupting tooth; and group C: having all 20 primary teeth.

## Data Availability

The datasets generated during and/or analyzed during the current study are not publicly available due to the privacy of patients’ information, but they are available from the corresponding author on reasonable request.
